# The effects of emotional distress on attentional bias toward cigarette warnings according to smokers' anxiety levels

**DOI:** 10.3389/fpsyg.2024.1411747

**Published:** 2024-12-16

**Authors:** Younji Jung, Jang-Sun Hwang, Jang-Han Lee

**Affiliations:** ^1^Department of Psychology, Chung-Ang University, Seoul, Republic of Korea; ^2^Department of Advertising & PR, Chung-Ang University, Seoul, Republic of Korea

**Keywords:** anxiety, cigarette warnings, eye-tracking, emotional distress, smoker

## Abstract

Anxiety is related with the substance use, including cigarette smoking. Avoidance is one of the strategies smokers with anxiety adopt to manage negative affect, which can be contradictory to a strategy of cigarette warnings that is used to induce negative affect to change smoking behaviors. Therefore, this study examined whether smokers' anxiety levels decrease their attentional biases toward cigarette warnings, especially in response to emotional distress. High-anxiety (*n* = 60) and low-anxiety (*n* = 60) smokers were randomly assigned to either a stress condition that utilized the PASAT-C task (Paced Auditory Serial Addition Task-Computer version) or a controlled condition. With the eye-tracking task that involved viewing 8 visual stimuli of cigarette packs composed of warnings and brandings, time to first fixation and fixation duration to warnings compared to brandings were measured both pre and post conditions. The results revealed that high-anxiety smokers detected warnings faster after stress conditions while low-anxiety smokers showed the consistent time to first fixation on warnings. In terms of fixation durations, high-anxiety smokers showed hypervigilance toward warnings that are considered to be a threat, but low-anxiety smokers showed avoidance under stress conditions, particularly toward social-focused warnings. These results indicate that high-anxiety smokers are more vulnerable to emotional distress and have an attentional bias toward fear appeals. Despite hypervigilance, they had greater psychological reactance toward warnings that the conflict between avoidance and hypervigilance might have contributed to, so the effectiveness of fear appeals may be limited regardless of the increased fixation duration.

## 1 Introduction

Anti-smoking efforts with communication tools have been widely devoted throughout the world for more than 30 years. Among various public and social communication campaigns executed by public organizations including governments, anti-smoking campaigns are the top budget-spent communication activity. For example, a recent anti-smoking advertising campaign executed by the Korean government, the so-called “*no-dam”* (meaning “no cigarette”), spent over 20 billion dollars per year (Kim and Choi, 2020).

The majority of previous studies focused on the effectiveness of message strategies used in anti-smoking advertising campaigns. Since increasing amounts of budgets have been spent on mass media advertising, it is critical to find an effective message strategy for various targets including heavy-, light-, and prospective smokers. Specifically, many studies investigated the effectiveness of fear appeal vs. positive appeals, and the results are somewhat controversial. Nonetheless, research in the communication field dealing with a communication tool for anti-smoking have been rather neglected. It is the warning message on cigarette packs exposed to all cigarette consumers, which should receive more attention.

As a communication tool for anti-smoking, Graphic Health Warnings (GHWs) on cigarette packs have been widely used in Korea as well as other leading countries. In fact, GHWs on cigarette packs have been adopted in Korea since 2016, but their effectiveness in terms of declining smoking rate is still in question. According to the 2020 National Health and Nutrition Examination Survey, the percentage of current male smokers over 19 years old has declined from 40.7% to 34.0% since 2016 whereas that of female smokers has slightly increased from 6.4% to 6.6% (KBS, 2022). The number seems promising at least for male smokers, yet the fact that the decrease in their rate was already occurred from 48.3% to 40.7% since 2010 as well as the fact that there was a considerable rise in the cost of cigarettes in 2017 has prompted researchers to keep on seeking after the answer.

Numerous studies attempted to assess the effectiveness of cigarette warnings with different self-reported questionnaires and behavioral tasks, one of which is the eye-tracking task measuring smokers' avoidance of GHWs. Studies with GHWs drew more attention than text-only warnings (Strasser et al., 2012) appeared to establish their effectiveness, but others disclose that smokers refuse to engage with health warnings by avoiding them.

The studies that measured visual attention between tobacco health warnings and branding (Maynard et al., 2014; Munafò et al., 2011), as well as the studies that measured visual attention between tobacco health warnings and neutral stimuli using various tasks (Loeber et al., 2011; Jang and Yoon, 2021), revealed that smokers show attentional avoidance toward warnings. In particular, provocative visual stimuli, such as diseased or damaged body parts, were correlated with the intention to avoid health warnings (Korkmaz et al., 2024; Sutton et al., 2019). However, other studies found that visual attention to warnings with ill bodies was greater (Sidhu et al., 2021) and more attention was paid to health warnings over branded images (Byrne et al., 2018). Even the studies of systematic review taking various factors into consideration yielded different conclusions on the effectiveness of GHWs on cigarettes (Monárrez-Espino et al., 2014; Noar et al., 2016). Visual attention to health warnings is often assessed with the intention to quit (Park et al., 2020), where as another study showed that smokers who reported to avoid health warnings via questionnaires found them rather more believable and were more likely to quit (Cannoy et al., 2023). Therefore, the avoidance of health warnings on cigarette packs could be a significant factor in assessing how they perceive their smoking behaviors.

In order to explore the avoidance of GHWs, how it is related to other characteristics of smokers that could possibly attribute to avoidant behaviors also needs to be investigated. In the current study, anxiety was assumed to have a pivotal role in avoidance. There are several reasons why smokers smoke, one of which is to control negative affect (Guthrie et al., 2001; Stevens et al., 2005), also supported by the Motivational Model of Substance Use that explains the motivation of substance users as the coping motive to manage negative emotions and the conformity motive to reduce negative social experiences (Studer et al., 2016). Among negative affects, anxiety was found to be strongly associated with nicotine dependence (Sonntag et al., 2000). Maladaptive attempts to manage negative affects with smoking rather than employing adaptive strategies were seen in anxious smokers (Buckner et al., 2020) who were more likely to smoke when they were anxious (Henker et al., 2002). Many smokers may frequently use cigarettes to decrease negative affect, and those with higher levels of anxiety may be more dependent on cigarettes to decrease such affects.

Anxiety sensitivity, a trait that one fears the consequences of anxiety, has been also associated with substance use for coping with negative affect relating to smoking and drinking (Guillot et al., 2016). The study examining the association between anxiety and smoking showed that smoking increases anxiety sensitivity and the probability of experiencing panic attacks by four times and that the onset of smoking precedes anxiety-related symptoms (Breslau et al., 2001). A recent systematic review investigating the role of anxiety in smoking behavior concluded that people with anxiety are more likely to be smokers, but the evidence of its association with onset, severity, and quitting was relatively weak (Garey et al., 2020).

Smokers' anxiety and their coping strategies might prompt avoidance of health warnings. Research has focused on avoidance, because experiential avoidance, a cognitive-emotional regulation process of an individual who does not want to experience uncomfortable thoughts, emotions, and images (Bakhshaie et al., 2015), plays an important role in maintaining substance addiction (Chawla and Ostafin, 2007). One of the factors that maintain smoking with higher levels of anxiety is false safety behavior, which refers to the behavior that is used to reduce anxiety instantly in response to false threats, like avoiding the circumstance (Schmidt et al., 2012). However, this behavior appears to maintain or even increase anxiety in the long term, due to the loss of an opportunity to verify whether threats are false or not (Piccirillo et al., 2016). There is a term called avoidance and inflexibility to smoking (AIS), which is a smoking-specific experiential avoidance that reflects the proneness to avoid distressing states including thoughts and feelings through smoking (Gifford and Lillis, 2009).

This kind of coping strategy that smokers with a higher level of anxiety use contradict the purpose of tobacco health warnings: one is to relieve negative affect and the other is to evoke negative affect. Tobacco health warnings in cigarette packs are categorized as fear appeals that have the purpose of changing the target behavior by causing negative affect about it (Witte, 1994), so they aim for smokers to change smoking-related intentions by making them feel bad about their smoking behaviors. A study shows that GHWs do increase negative affect (Skurka et al., 2018) and the stronger GHWs elicit emotions, the more effective they are considered (Shi et al., 2017). However, they do not always serve their purposes, because negative affect has different impacts on people and there are other ways to reduce negative affect rather than just conforming to the threatening messages. According to the Terror Management Health Model, when a threatening stimulus such as tobacco health warnings emerges, people react in a defense mechanism to reduce perceived vulnerability to the threat by either enacting healthy actions or showing maladaptive avoidance reactions such as denying the threat, finding distractions, or procrastinating (Greenberg et al., 2000).

Anxiety and smoking show a high rate of co-occurrence, and smokers with anxiety report using smoking as a way to alleviate negative affect (Guillot et al., 2016), but how anxiety works in relation to how smokers perceive health warnings is rarely known. There are many studies that have studied the relationship between anxiety and smoking, but studies have rarely examined the relationship between anxiety of smokers and avoidance of health warnings in cigarette packs. Smokers are exposed to health warnings daily, so it would be worthwhile to investigate how the level of anxiety affects visual attention toward health warnings, and what patterns anxious smokers exhibit when their strategies to control negative affect conflict. Smokers trying to cope with negative affects by smoking may have greater visual avoidance of GHWs in cigarette packs as well as avoidance toward negative affect in life.

Besides anxiety, there are various individual characteristics that influence the reaction to GHWs. Avoidance toward GHWs may be due to the wear-out effect by over-consumption of or adaptation to disgusting and aversive images. Although GHWs in Korean cigarette packs have been renewed every 24 months, the similarities of images and messages are kept as they do in other countries. The effect of repeated exposure to GHWs was seen to be distinct, depending on smokers' characteristics like disengagement belief and the number of cigarettes they smoked (Dijkstra and Bos, 2015), or even positive (Rooke et al., 2012; Sidhu et al., 2022). Avoidance may also be due to psychological reactance, which was seen to be inversely associated with the perceived risk of pictorial warnings (Hall et al., 2018), but another study found that stronger reactance resulted in more quitting attempts despite reporting the likelihood of avoiding warnings (Cho et al., 2016). Trait worry (Farris et al., 2016a) and lower health literacy (Quisenberry et al., 2018) were associated with greater visual attention toward GHWs.

Another characteristic of people with anxiety is distress intolerance. Smokers with the tendency to avoid distressing states may lack distress tolerance. Distress intolerance indicates the degree of unwillingness to tolerate negative affect, which appears to be related to the maintenance of smoking (Farris et al., 2016b). A study explored that it is related to the severity of smoking status (Leyro et al., 2011), while another one didn't (Veilleux and Skinner, 2020). Studies demonstrated that smokers with a lower threshold of tolerating distress had a shorter period before relapsing after quitting attempts (Brown et al., 2002). This study has also explored its association with smoking behavior because it is significant in terms of managing negative affects as it was found to be linked to smoking to relieve negative affect (Trujillo et al., 2017). A previous study (Karekla et al., 2017) induced emotional distress increased smokers' desire to smoke, and the relationship between the two was explained by an increase in negative effects. Therefore, this study used the PASAT-C task that is known to induce emotional distress and psychological stress to create an environment to measure distress intolerance and to see how emotional distress affects smokers' visual attention toward cigarette warnings.

According to the Transtheoretical Model, people actively avoid information if they are not prepared to change their behavior about it (Prochaska and DiClemente, 1986). In addition, according to the Elaboration Likelihood Model, motivation, ability, and opportunity must work together to process information deeply enough to induce a change of behavior (Cacioppo et al., 1986). Therefore, stressful events will decrease the motive to quit or increase cravings for smoking in anxious smokers with the tendency of managing negative affects by smoking. Moreover, their ability to quit smoking decreases as they rely on smoking to manage under stressful situations. When people face threat stimuli, their attention bias to them increases when they feel they are in control of the threat, but the opposite effect appears when feeling they are not in control (Notebaert et al., 2020). Therefore, when faced with stressful situations where negative affects increase, smokers may have a higher desire to smoke, and their control over the threat of aversive graphic health warnings is reduced, which is expected to further increase their avoidance of GHWs.

The aim of this study was to examine (1) whether smokers with higher levels of anxiety show greater visual avoidance toward tobacco health warnings in cigarette packs, (2) greater distress intolerance, and (3) whether such avoidance increases under stress conditions as anxiety and smoking cravings increase. The study examined how anxiety prompting substance use and avoidance is intertwined with visual attention to health warnings, distress intolerance, and cravings under stress and control conditions. Visual attention was measured using an eye-tracking task, and time to first fixation and fixation duration/count were compared. Distress tolerance was measured using the PASAT-C task, as a laboratory stressor that induces emotional distress, and the time until quitting the task was compared. This study has focused on the level of anxiety that was sustained over at least a week and is more symptomatic anxiety rather than trait anxiety. How various characteristics of smokers with higher levels of anxiety contribute to their visual attention toward cigarette warnings, especially in the presence of emotional distress will give a better understanding of smokers, which will give better strategies to improve health warnings.

## 2 Materials and methods

### 2.1 Participants

Participants were 120 Korean daily cigarette smokers who were recruited through online and offline advertisements. Only daily smokers and those who use tobacco cigarettes, but not e-cigarettes, for more than 6 months were recruited to ensure they are exposed enough to health warnings in cigarette packs. Participants were eligible if they were older than 18 years old and had normal vision to be able to perform the eye-tracking task. Exclusion criteria included the presence of an intellectual problem assessed by the inability to give consent and follow the instructions of the behavioral tasks, especially the PASAT-C task.

In order to participate in the study, people had to complete an online screening survey containing three questionnaires: Beck Anxiety Inventory (BAI), Smoking Motivation Questionnaire (SMQ), and Anxiety Sensitivity Index-3 (ASI-3). BAI was the only criterion for screening to allocate participants into high-anxiety and low-anxiety groups. SMQ was included to disguise the screening criteria to prevent people from faking their condition to be selected to participate in the study.

The cutoff score of BAI for the group of high-anxiety smokers was determined to be 22 based on the previous studies (Cho, 2020; Hong and Park, 2016), as this cutoff score is more frequently used in Korea, but may vary depending on ethnicity and can range from 7 to 26 (Bardhoshi et al., 2016). Those who got 22 or higher were categorized as high-anxiety smokers and those who got 16 or lower were categorized as low-anxiety smokers. Those who fell into the range between 17 and 21 were not included to make a clear distinction between groups. A total of 60 people for each group were selected among 278 people who applied for the experiment: 60 smokers with a higher level of anxiety and 60 smokers with a lower level of anxiety.

This study contains a condition-forming stage, in which two different conditions, stress condition and control condition, are formed in order to see the effect of stress on two types of groups. Participants in each group were randomly assigned to either a stress or a control condition. Therefore, there were a total of four different groups, each group consisting of 30 smokers: 30 high-anxiety smokers under a stress condition, 30 high-anxiety smokers under a control condition, 30 low-anxiety smokers under a stress condition, and 30 low-anxiety smokers under a control condition.

The sample size for the present study was calculated using the G^*^power 3.1 program (Faul et al., 2009). A total of 76 subjects are adequate for a design with repeated-measure analysis of variance (ANOVA), an alpha error probability of 0.05 (two-tailed), a power of 0.95, and a medium effect size ($\eta_{p}^{2}$ = 0.25). To find out the difference in visual attention to health warnings between high-anxiety smokers and low-anxiety smokers who are in either a stress or a control condition.

### 2.2 Measurement

#### 2.2.1 Self-report questionnaires

##### 2.2.1.1 Demographics questionnaire

Participants were asked about gender, age, and smoking history. Those questions consist of the number of cigarettes they use per day, the age at which smoking began, and the most preferred brands of cigarettes.

##### 2.2.1.2 Beck anxiety inventory

The BAI was developed to measure the degree of anxiety subjects have as it measures the frequency of anxiety symptoms (Beck et al., 1988). In this study, the Korean version of the BAI validated by Yook and Kim (1997) was used. The BAI is comprised of 21 items that are divided into cognitive, emotional, motivational, and physical components of anxiety. It uses a 4-point Likert scale (0 = “not at all,” 1 = “a little”, 2 = “much,” 3 = “very much”). Scores can be distributed from 0 to 63 points. A score between 22 and 26 points is considered as being in the state of having anxiety, a score between 27 and 31 as being in the state of having severe anxiety, and a score of 32 or higher as being in the state of having very severe anxiety (Hong and Park, 2016). The higher the total score, the higher the anxiety the person experiences. The BAI was used in this study to classify smokers into two groups that are a high-anxiety and a low-anxiety group. The cutoff score was 22 as mentioned above. In the validity study, Cronbach's α was 0.91, and in this study, Cronbach's α was 0.92.

##### 2.2.1.3 Anxiety sensitivity index-3

The ASI-3 was developed to measure the level of sensitivity toward anxiety, in other words, the fearfulness of symptoms of anxiety (Taylor et al., 2007), and its Korean version validated by Lim and Kim (2012) was used in this study. It consists of 18 items, 6 items for each subscale of physical, cognitive, and social concerns. ASI-3 is measured using a 5-point Likert scale (0 = “very little,” 1 = “a little,” 2 = “somewhat,” 3 = “much,” 4 = “very much”) and its score can be distributed from 0 to 72. The higher the score, the more sensitive individuals are to the symptoms of anxiety. As anxiety sensitivity is also known to be associated with smoking to alleviate anxiety (Zvolensky et al., 2005), this study intends to find out its association. In the validity study, Cronbach's α was 0.87, and in this study, Cronbach's α was 0.94.

##### 2.2.1.4 Smoking motivation questionnaire

The SMQ was developed by Spielberger et al. (1983a) to measure smokers' motives for smoking, and the Korean version that was revised in regard to Korean culture by Lee and Han (1995) was used in this study. This scale is comprised of 31 items with 5 subscales: 12 items for management of negative affects, 6 items for rest and boredom, 5 items for intellectual stimulation and curiosity, 2 items for social attraction, and 6 items for habitual smoking. All items were available for participants to have enough options to choose for smoking motive, but only 12 items for management of negative affects were calculated for the score. This SMQ is a 4-point Likert scale (1 = “not at all,” 2 = “rarely,” 3 = “somewhat,” 4 = “very much”), so the score can be distributed from 12 to 48. In this study, the SMQ was used to clarify any effect that smokers' motives have on visual attention toward health warnings. In the study that used this scale (Kim et al., 2012), Cronbach's α was 0.91, and in this study, Cronbach's α was 0.90.

##### 2.2.1.5 Fagerstrom test for nicotine dependence

The FTND, developed by Heatherton et al. (1991) and translated into Korean by Ahn et al. (2002) was used to measure the degree of nicotine dependence. This scale is comprised of 6 items, its score ranging from 0 to 10. A score of 3 or lower is considered to be mild dependence, a score from 4 to 6 to be moderate dependence, and a score of 7 or higher to be severe dependence. The FTND was used to determine whether smokers' dependence on nicotine is associated with other measurements. In the validity study, Cronbach's α was 0.69, and in this study, Cronbach's α was 0.55.

##### 2.2.1.6 Self-efficacy in smoking cessation

This scale was developed by Velicer et al. (1990) to measure an individual's ability or confidence to control smoking desire in certain situations and was translated into Korean by Kong and Ha (2013). It is comprised of 9 items, using a 5-point Likert scale (1 = “not tempted at all,” 2 = “not tempted,” 3 = “moderately tempted,” 4 = “a little bit tempted,” 5 = “very tempted”). The score ranges from 9 to 45, and the higher the score, the more self-efficacy one has in smoking cessation as the scores are calculated reversely. This study used this scale to see how self-efficacy in smoking cessation affects visual attention toward health warnings, as its messages can be less disturbing when one has confidence in conducting the healthy action. In the study of Kong and Ha (2013), Cronbach's α was 0.95, and in this study, Cronbach's α was 0.69.

##### 2.2.1.7 Quitting smoking contemplation ladder

The QSCL is a tool to measure smoking cessation intention, developed by Biener and Abrams (1991). This scale consists of only 1 item, its score ranging from 1 to 10 (1 = “I enjoy smoking and have decided not to quit smoking for my lifetime. I have no interest in quitting,” 10 = “I have quit smoking and will never smoke again”). As this scale has only one item, it was translated into Korean to be used in this study. In the study of Sillero-Rejon et al. (2021), 1 to 4 points were classified as those who did not intend to quit smoking and 6 to 8 points were classified as those who have the intention to quit. In this study, QSCL is used to confirm whether the intention to quit smoking is associated with visual attention to health warnings, as the intention to quit may be related to willingness to accept the health warning messages.

##### 2.2.1.8 Personalized psychological flexibility index

The PPFI was developed to measure psychological flexibility by Kashdan et al. (2020) and the Korean version was validated by Park (2022). Psychological flexibility is the ability to flexibly respond to situations in the process of pursuing goals in life that accompanies pain or discomfort. In other words, it is the willingness to endure an uncomfortable internal state in order to pursue a life goal, and a lack of psychological flexibility is considered experiential avoidance (Park, 2022). PPFI consists of three subscales: avoidance, acceptance, and utilization which have 5 items each. In this study, only two subscales that are avoidance and acceptance are used, as this study is interested in how avoidance pattern in life is associated with other measurements. The first three questions are scored, but for setting the mood to consider their life goals. It is a 7-point Likert scale, ranging from 10 to 70 points (1 = “strongly disagree,” 2 = “disagree,” 3 = “slightly disagree,” 4 = “neither disagree nor agree,” 5 = “slightly agree,” 6 = “agree,” 7 = “strongly agree”). The items in the avoidance subscale are reverse-scored, so the higher the score, the higher the psychological flexibility. In the validity study, Cronbach's α was 0.72, and in this study, Cronbach's α was 0.79.

##### 2.2.1.9 Scale of predicting risk behaviors

Risk perception is an evaluation tool developed by Witte (1996) to measure one's evaluation on the possibility of a specific situation or issue. This study used the Korean version that was revised and modified by Yang (2017). The scale is comprised of 6 items with subscales of sensitivity and severity. It is a 5-point Likert scale, ranging from 5 to 30 (1 = “not likely at all,” 2 = “not likely,” 3 = “moderately,” 4 = “likely,” 5 = “very likely”). This study used this scale in order to see how smokers perceive the risk of smoking and how health warning affects their perception. In the study of Yang (2017), Cronbach's α was 0.86, and in this study, Cronbach's α was 0.72.

##### 2.2.1.10 Psychological reactance scale

This scale was developed by Hong and Page (1989) to measure the degree of psychological reactance toward health warnings and Kim (2015) translated it into Korean and modified it. It is comprised of 8 items with a 5-point Likert scale (1 = “not at all,” 2 = “not likely,” 3 = “less likely,” 4 = “moderately,” 5 = “likely”), ranging from 8 to 55. The higher the score, the higher the psychological reactance. This study used this scale to see how psychological reactance toward tobacco health warnings is associated with visual attention to them. In a previous study, quit intention was positively associated with risk perception and negatively associated with psychological reactance, and risk perception and psychological reactance showed a negative association (Park and Park, 2021). In the validity study (Hong et al., 1994), Cronbach's α was 0.90, and in this study, Cronbach's α was 0.87.

##### 2.2.1.11 State-trait anxiety inventory

The STAI-X-1 was developed by Spielberger et al. (1983b) to measure the degree of anxiety subjects feel at a particular time. This study used the one that was translated into Korean and validated by Kim and Shin (1978). It is comprised of 20 items and a 4-point Likert scale, ranging from 20 to 80 (1 = “not at all,” 2 = “somewhat,” 3 = “moderately so,” 4 = “very much so”). As it measures the frequency of anxiety symptoms (Beck et al., 1988). The higher the score, the more anxious one is at the moment. This measurement is different from BAI in that it measures the level of anxiety one feels at the moment, not the level of anxiety one feels generally. It is used to measure the change in the level of anxiety of smokers during the experiment as they are assigned to either a stress condition or a control condition. Measurement takes place before and after the assigned condition. In the validity study, Cronbach's α was 0.84, and in this study, Cronbach's α was 0.93.

##### 2.2.1.12 Craving-visual analog scale

The VAS was first developed by DeLoach et al. (1998) to evaluate pain intensity, but in this study, it is used to evaluate the changes in craving for smoking. It is one question of “How much do you desperately want to smoke at this moment?” in Korean and participants were asked to mark on a 10 cm line (Jung, 2014). The line is presented with the marks of 0 on the left side with “I do not want to smoke at all” and 100 on the right side with “I want to smoke very badly.” Participants' answers are measured with a ruler and the score ranges from 0 to 100, 1 mm being 1 point. This scale was used to determine the changes in cravings of participants before and after stress or control conditions. As repeated measurements can offer baseline cravings, smokers were not asked to change their smoking habits before participating in the experiment.

#### 2.2.2 Bahavioral tasks

##### 2.2.2.1 The eye-tracking task

The means to analyze visual attention toward tobacco health warnings is through an eye-tracking device that allows capturing the detailed eye movement on visual stimuli (Duchowski and Duchowski, 2017). This task measures visual attention to the areas of interest (AOIs) that the experimenter assigns. There are three parts in the front section of cigarettes: a GHW, a text message, and a branding. In this study, AOIs were assigned only to a graphic health warning and a branding. The middle part of text messages was excluded to avoid the overlap among AOIs as they are very close and to promote clear analysis.

The health warning pictures are replaced every 2 years, and the current ones have been applied since December 23, 2020. Therefore, they have been exposed to smokers for more than a year and a half to date. To minimize the effect of repeated exposure on avoidance toward GHWs, the ones that are being used in other countries were included, so 10 pictures that are currently in cigarette packs in Korea and 10 pictures from cigarette packs of other countries were used for visual stimuli. The foreign health warnings were selected to match the messages of Korean health warnings. Both Korean health warnings and foreign health warnings are currently used in cigarettes and their pictures are open to the public. In addition, warning pictures were categorized into two types: health-focused ones, indicating damage to the body part, and social-focused ones, indicating the consequences of affecting social relations (Park et al., 2020; Shen, 2011). Both types were designed to be exposed at the same rate. Since there are two types of GHWs from two different sources, the number of stimuli that were used for each trial was eight, two from each category. The four categories are Korean Health-focused GHWs, Korean Social-focused GHWs, Foreign Health-focused GHWs, and Foreign Social-focused GHWs. The warning pictures were combined randomly with 18 cigarette brands. Measurements by eye-tracking were measured twice before and after the allocated condition, so eight cigarette packs with GHWs were repeated twice. For the second trial of the eye-tracking task, half of the eight cigarette packs were the same ones used in the previous trial for consistency, but the rest were the new sets of combinations to minimize the effect of repeated exposure and boredom. A total of six sets were produced and randomly applied to participants regardless of their groups.

**Figure 1:**
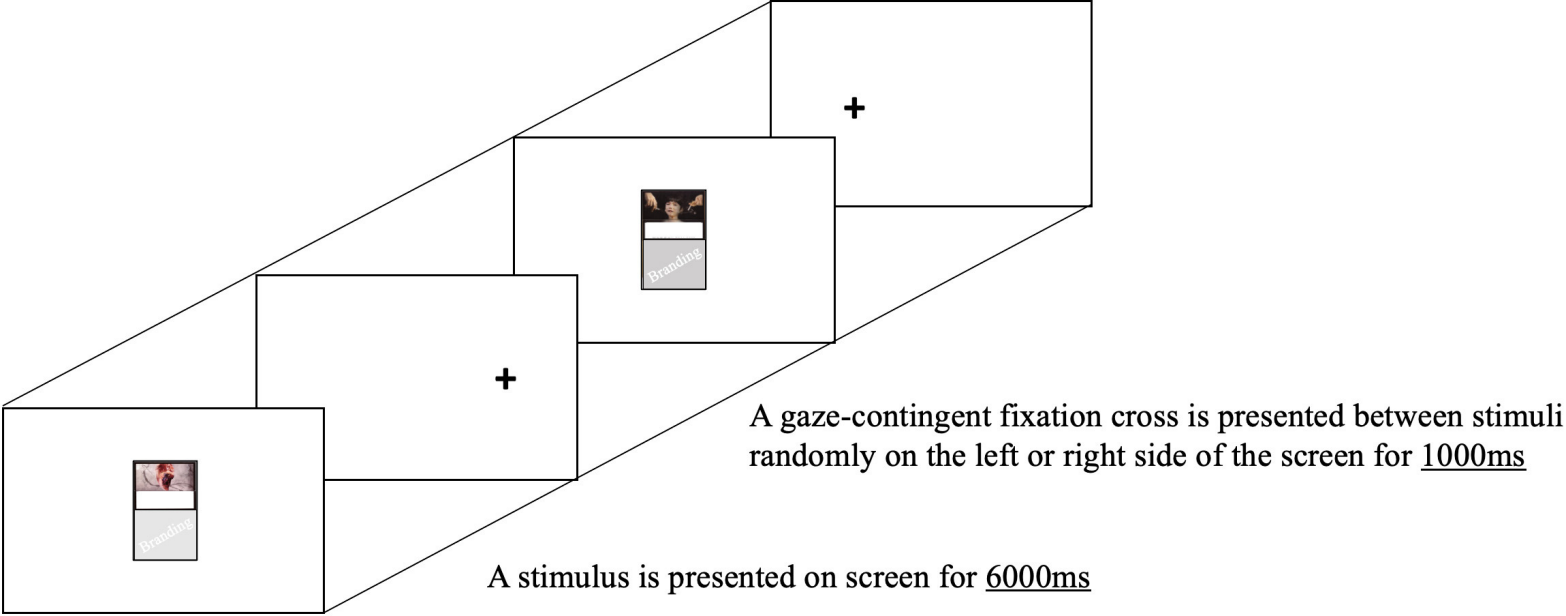
Trial example of the eye-tracking task.

This task was a free-viewing task, so participants were asked to watch cigarette packs (see Figure 1). They were informed that the cross appears randomly either on the left or right side of the white screen for 1,000 ms to ensure a more accurate time to first fixation by preventing random gaze fixation (Sillero-Rejon et al., 2021). Then, the cigarette pack visual stimulus appears on the white screen for 6,000 ms in the size of the actual cigarette pack, which is 55 mm × 88 mm. Since it is a free viewing task, practice is not given separately, and participants in the study are asked to look at the screen freely as they would usually see cigarette packs without any intention. The task was intended to compare the time to first fixation and the fixation duration at each AOI, and the fixation count at each AOI.

##### 2.2.2.2 The paced auditory serial addition task-computer version

**Figure 2:**
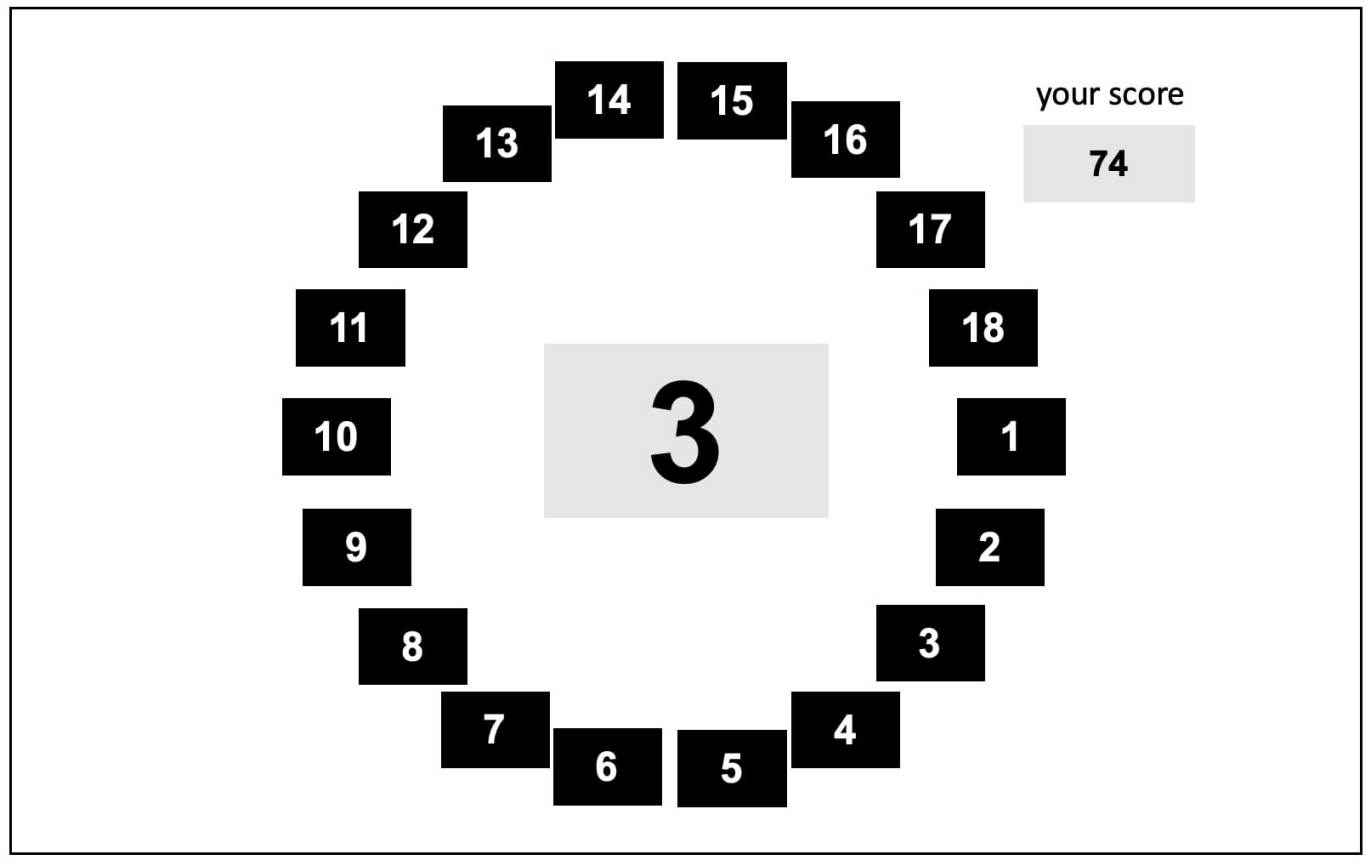
Trial Example of the PASAT-C Task. PASAT-C Task: Paced Auditory Serial Addition Task-Computer version.

Smokers were randomly assigned to either a stress condition or a control condition to confirm the effect of emotional distress on visual attention, state anxiety, and smoking craving. In this study, the PASAT-C task was selected as a laboratory stressor to induce emotional distress, as this task was validated to provoke stress in the form of anxiety, irritability, and frustration in both clinical and non-clinical samples (Bornovalova et al., 2008; Lejuez et al., 2003). Among other tasks that induce stress, this task is specifically chosen as the induced emotional distress is not limited to social anxiety. The PASAT-C task is a task to correctly answer with the sum of the last two numbers while numbers are presented constantly (see Figure 2). For example, if 3 and 7 are presented, you are supposed to click 10 among the blue numbers presented on the screen in the circle. Then, if another number, 8, is presented, you are supposed to forget the first number 3, and add the last two numbers, 7 and 8, so click 15 on the screen and then 12 if 4 is presented. This task has been demonstrated that it has a stress-inducing effect as a difficult task to forget the old data and utilize new data at a speedy pace (Karekla et al., 2017). It consists of three levels, and as the level increases, the speed at which the number is presented increases so does the difficulty. However, at the third level, participants are given the option to quit at any time they want as the “QUIT” button appears. In addition to its difficulty, the task gives annoying sounds whenever participants get wrong. The screen also provides the number of correct answers, which gives participants the pressure to do a better job. This task begins after the exercise and takes 10 min until the second level and 10 more minutes until completion. At the third level, distress intolerance is measured by the time it takes participants to press the “QUIT” button. In addition, the number of correct answers also allows us to compare the performance between high-anxiety smokers and low-anxiety smokers. The time to press the “QUIT” button in milliseconds, which is latency, signifies distress tolerance. If one completes the task without pressing the “QUIT” button, latency will be 600,000 ms as the task after level 3 will take 10 min.

Under control conditions, a task is given to pass some time between repeated measurements of eye-tracking tasks, anxiety, and smoking cravings. It is a simple and easy copying task to draw simple figures and write sentences, which was intended not to cause any stress or anxiety, but at the same time to use cognition and be distracted from the previous measurements. The figure is a simple figure in which two or three circles, squares, and lines are overlapped, and the sentence was extracted from a newspaper, the content of which is not related to smoking. Participants in the experiment are informed that they do not have to do the task perfectly as it is intended to see normal cognitive function and healthy motor ability.

### 2.3 Apparatus

For the eye-tracking task, a 15-inch PC computer attached with Eye Tracker TX300 was used. The tasks were presented with a resolution of 1920^*^1080 through version 3.4.8 of tobii studio program. Participants were 30 cm to 40 cm away from the screen while seated on a stable chair. They were able to position themselves in the middle of the camera's field, with the help of tobii studio's showing their eye position before calibration.

For the PASAT-C task and other self-reported questionnaires, a 13-inch Intel Core i5 MAC laptop on a separate desk was used. Participants were allowed to use either a wireless mouse or trackpad as they preferred.

### 2.4 Procedure

The experiment was approved by Chung-Ang University Institutional Review Board (IRB No. 1041078-202209-HR-217). It was a single session and was conducted in a room with the equipment at Chung-Ang University. All participants were informed about the experiment and those who signed the consent form proceeded with it.

All participants were asked to complete self-reported questionnaires of FTND, Self-efficacy in Smoking Cessation, QSCL, PPFI, STAI-X-1, and Craving-VAS. Then, participants moved to the desk where the eye-tracking device was and positioned themselves to be appropriate to perform the task. The viewing started with a calibration exercise where their eyes had to follow the moving red dot. They were informed that they had to fixate on the cross sign that appears either on the left or right side before a cigarette image and view the cigarette images freely without any intention.

Participants who were assigned to a stress condition were then instructed on how to do the PASAT-C task. After doing the exercise, they proceeded with levels 1 and 2 without intervention. This lasted about 10 min. After completing level 2, they were instructed that the difficulty was about to go up at level 3, but they could quit anytime they wanted by pressing the “QUIT” button, and if they were to proceed till the end, the task would take the additional 10 min.

Participants who were assigned to a control condition were instead instructed with a copying task. They were asked to copy 4 figures and 4 sentences and were provided with a pen and paper. They were informed that they could take time as long as they wanted to complete the task.

After the condition-forming stage, all participants were asked to complete self-reported questionnaires of STAI-X-1 and Craving-VAS one more time as well as the eye-tracking task. After the eye-tracking task, self-reported questionnaires of Scale of Predicting Risk Behaviors and Psychological Reactance Scale were completed. These two questionnaires were done lastly because they involve questions on how people perceive the cigarette warning and thus may impact the free-viewing task.

The experiment took approximately 40 min. Participants were debriefed and reimbursed 15,000 won.

### 2.5 Data analysis

To investigate the difference in demographic and psychological characteristics between high- and low-anxiety groups, independent samples *t*-tests of age, gender, the average number of cigarettes people smoke per day, age of first smoking, ASI, SMQ, FTND, Self-efficacy in Smoking Cessation, QSCL, PPFI, Scale of Predicting Risk Behaviors, and Psychological Reactance Scale were conducted.

For the eye-tracking task, to investigate the differences in the gaze patterns that are time to first fixation and fixation duration between high- and low-anxiety groups, independent samples *t*-test of the measurements from the first trial of the eye-tracking task was conducted, but to investigate the difference in changes of those gaze patterns between high- and low-anxiety groups under stress and control conditions, a 2 (anxiety: high, low) × 2 (condition: stress, control) × 2 (time: before, after) three-way mixed ANOVA was conducted. Since this study was interested in smokers' attention toward GHWs in regard to the attention toward branding and the absolute time was not standardized, fixation durations on warnings are calculated by the ratio of visual attention toward GHWs to visual attention toward GHWs and branding [fixation duration on warnings/(fixation duration on warnings + fixation duration on branding)].

For self-reported measurements that were used to see the change between before and after condition-forming state, a 2 (anxiety: low, high) × 2 (condition: stress, control) two-way ANOVA was conducted. Since this study did not try to control smokers' normal smoking behaviors and smokers may have different cravings depending on the time of smoking and their circumstances, a simple calculation of subtracting the baseline measurements from the later measurements was used instead of the analysis by repeated measure ANOVA to demonstrate the difference in the direction of changes. The same analysis was used for the change in STAI-X-1.

For the PASAT-C task, only the groups that were under stress conditions were included for analysis, so independent samples *t*-tests of distress tolerance and correct answers were conducted.

Since there are only two levels in each factor, no *post-hoc* test was performed. All statistical analyses were performed using IBM SPSS Statistics for Windows version 26.

## 3 Results

### 3.1 Demographics and group characteristics

The descriptive characteristics of participants are shown in Table 1. These include demographic characteristics, smoking history, and BAI. They were compared by the one-way ANOVA among four groups. The results showed that there were no significant differences in mean age [*F*_(3,116)_ = 1.89, *p* = 0.14], the number of cigarettes per day [*F*_(3,116)_ = 1.03, *p* = 0.38], and the age of first smoking [*F*_(3,116)_ = 0.99, *p* = 0.40]. These results indicate that the groups did not differ in demographic characteristics and smoking history except for the BAI that was the criteria for allocating the participants into high-anxiety and low-anxiety groups [*F*_(3,116)_ = 190.83, *p* < 0.001, $\eta_{p}^{2}$ = 0.83; *t*_(118)_ = 23.43, *p* < 0.001, MD(Mean Difference) = 21.87].

**Table 1:** Group characteristics and differences between high-anxiety and low-anxiety smokers.

	**High-anxiety**		**Low-anxiety**		**Test statistics (*F, t*)**
	**Stress (*****n** =* **30)**	**Control (*****n** =* **30)**	**Stress (*****n** =* **30)**	**Control (*****n** =* **30)**	
Age (years)	22.97 (2.87)	23.90 (2.89)	23.40 (3.72)	24.90 (3.67)	1.89
Sex (male %)	57 (50.40)	50 (50.90)	77 (43.00)	73 (45.00)	2.21
Number of cigarettes	10.63 (9.70)	7.93 (5.67)	9.82 (4.62)	10.18 (3.97)	1.04
Age of first smoking (years)	17.70 (2.17)	18.42 (2.80)	18.73 (1.87)	18.33 (2.59)	0.99
BAI	30.00 (4.48)	26.97 (6.18)	6.23 (4.35)	7.00 (4.90)	190.83^***^
ASI-3	35.22 (15.44)		12.28 (8.17)		10.17^***^
SMQ	42.90 (5.50)		37.88 (7.74)		4.09^***^
FTND	2.65 (2.19)		2.38 (1.64)		0.76
Self-efficacy in Smoking Cessation	16.15 (4.54)		18.53 (4.11)		−3.02^**^
QSCL	4.45 (1.55)		4.67 (1.48)		−0.78
PPFI	41.07 (10.15)		48.03 (6.71)		−4.44^***^
Avoidance	22.50 (6.13)		17.62 (4.80)		4.86^***^
Scale of Predicting Behaviors	22.10 (4.19)		20.90 (3.07)		1.79
Psychological Reactance Scale	23.97 (9.01)		17.72 (6.53)		4.35^***^
**Eye-Tracking Task**					
Time to First Fixation on W (sec)	2.19 (1.79)		2.51 (1.84)		−0.95
Fixation Duration on W (%)	45.76 (22.39)		39.52 (21.50)		1.56

Mean (standard deviation); BAI, Beck Anxiety Inventory; ASI-3, Anxiety Sensitivity Index-3; SMQ, Smoking Motivation Questionnaire; FTND, Fagerstrom Test for Nicotine Dependence; QSCL, Quitting Smoking Contemplation Ladder; PPFI, Personalized Psychological Flexibility Index; Fixation Duration on W (%), fixation duration on warnings/(fixation duration on warnings + fixation duration on branding); Test Statistics (*F*), results of the one-way ANOVA; Test Statistics (*t*), results of the independent samples *t*-test. ^**^p < 0.01. ^***^p < 0.001.

### 3.2 Comparison between high-anxiety and low-anxiety smokers

In order to see if there is any distinguishable difference between high-anxiety and low-anxiety smokers, independent samples *t*-tests were conducted for this comparison.

#### 3.2.1 Self-reported measurements

All the self-reported measurements except for the repeated measurements that are ASI-3, SMQ (management of negative affect), FTND, Self-efficacy in Smoking Cessation, QSCL, PPFI, Scale of Predicting Behaviors, and Psychological Reactance Scale were compared to see if any of these measurements represent the characteristics of high-anxiety smokers (see Table 1).

As ASI-3 was directly related to anxiety, the anticipated significant difference was shown. The average score of ASI-3 was also higher in high-anxiety group [MD (Mean Difference) = 22.93, *t*_(118)_ = 10.17, *p* < 0.001]. FTND, QSCL, and Scale of Predicting Behaviors showed no significant differences, which means that high-anxiety smokers do not necessarily have higher nicotine dependence, lower intention to quit, or lower awareness of the consequences of smoking. On the other hand, for the subscale of management of negative affect in SMQ, the average score was significantly higher in the high-anxiety group [MD = 5.02, *t*_(118)_ = 4.09, *p* < 0.001], which tells us that high-anxiety smokers have greater motives of managing negative affect with smoking. The average score of Self-efficacy in Smoking Cessation was significantly lower in the high-anxiety group [MD = 2.38, *t*_(118)_ = −3.02, *p* < 0.01], which means that high-anxiety smokers have less confidence in controlling their smoking urges. The average score of PPFI was significantly lower [MD = 6.97, *t*_(118)_ = −4.44, *p* < 0.001] and the average score of the subscale of avoidance in PPFI was significantly higher [MD = 4.88, *t*_(118)_ = 4.86, *p* < 0.001] in the high-anxiety group, which means that high-anxiety smokers have lower psychological flexibility and greater avoidance toward pain or discomfort in life. The average score of Psychological Reactance Scale was significantly higher in the high-anxiety group [MD = 6.25, *t*_(118)_ = 4.35, *p* < 0.001], which means that high-anxiety smokers show more reactance toward GHWs.

#### 3.2.2 Gaze patterns

In order to examine the differences between high-anxiety and low-anxiety groups in the gaze patterns when smokers encountered cigarette packs, time to first fixation and fixation duration for the first trial of the eye-tracking task were analyzed using the independent samples *t*-test (see Table 1).

For the time to first fixation on warnings, there was no significant difference between the two groups [*t*_(118)_ = 0.95, *p* = 0.34]. For the percentage of the fixation duration on warnings compared to brandings, there was also no significant difference between groups [*t*_(118)_ = 1.56, *p* = 0.12]. Overall, high-anxiety smokers did not show different gaze patterns from those of low-anxiety smokers in terms of time to first fixation and fixation duration before the condition-forming stage.

#### 3.2.3 Distress intolerance and performance on PASAT-C task

For the analysis of distress intolerance and performance of the PASAT-C task, only the smokers who were assigned to stress conditions were included. Although there was no significant difference in distress tolerance between high-anxiety and low-anxiety smokers [*t*_(58)_ = 0.60, *p* = 0.55], there was a significant difference in performance [*t*_(58)_ = 2.54, *p* < 0.05] (see Table 2). Low-anxiety smokers performed better on a difficult task, but the time to endure a difficult task did not differ significantly.

**Table 2:** Results of PASAT-C task.

	**High-anxiety under stress condition (*n =* 30)**	**Low-anxiety under control condition (*n =* 30)**	**Test statistics (*t*)**
**PASAT-C task**			
DT (milliseconds)	348,842.77 (20,8434.73)	381,751.20 (216,956.33)	−0.60
Performance (number of correct answers)	247.83 (73.63)	314.53 (123.91)	−2.54^*^

Mean (standard deviation); PASAT-C Task, Paced Auditory Serial Addition Task-Computer version; DT, Distress Tolerance; Test Statistics (*t*), results of the independent samples *t*-test. ^*^p < 0.05.

### 3.3 Comparison of the effect of emotional distress on high-anxiety and low-anxiety smokers

The main analysis for this study is to examine how high-anxiety smokers react differently under a stress condition that induced emotional distress.

#### 3.3.1 Self-reported measurements

**Figure 3:**
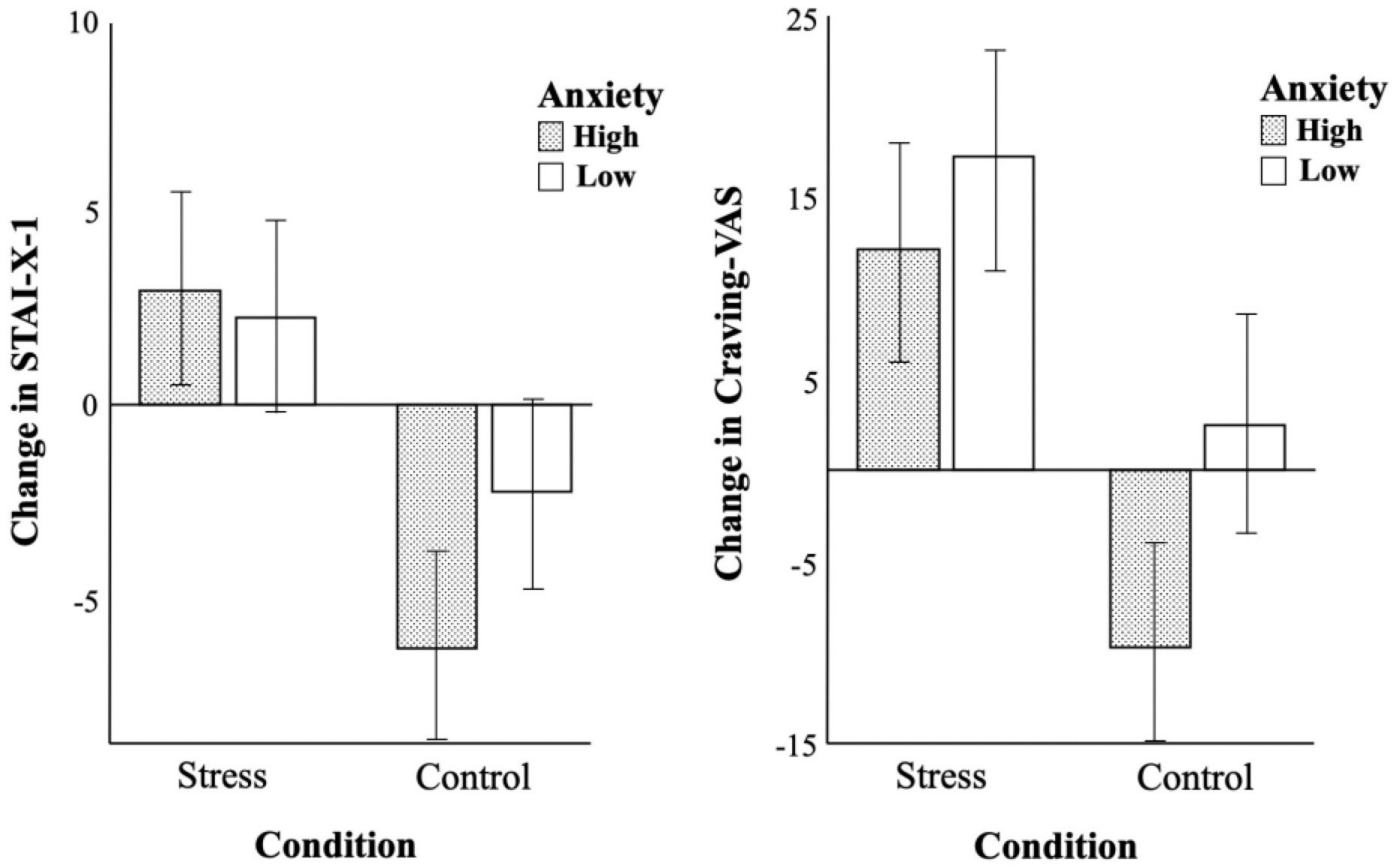
Comparison of changes in STAI-X-1 and craving-VAS before and after conditions between two groups.

For the self-reported measurements, changes in STAI-X-1 and Craving-VAS were analyzed using a 2 (anxiety: high, low) × 2 (condition: stress, control) two-way ANOVA, and results are shown in Table 3. For the changes in STAI-X-1 before and after conditions, there was a significant effect of stress [*F*_(3,116)_ = 30.55, *p* < 0.001, $\eta_{p}^{2}$ = 0.21], but not anxiety [*F*_(3,116)_ = 1.63, *p* = 0.21]. There was also no significant interaction between stress and anxiety [*F*_(3,116)_ = 3.36, *p* = 0.07]. For the changes in Craving-VAS before and after conditions, there were significant main effects of anxiety and stress [*F*_(3,116)_ = 8.14, *p* < 0.01, $\eta_{p}^{2}$ = 0.07; *F*_(3,116)_ = 34.96, *p* < 0.001, $\eta_{p}^{2}$ = 0.23], but no interaction [*F*_(3,116)_ = 1.45, *p* = 0.23]. State anxiety of both high-anxiety and low-anxiety smokers increased after stress conditions and decreased after control conditions, but the difference in state anxiety between stress and control conditions was bigger in high-anxiety smokers as the total height between conditions within each group is bigger in Figure 3, which means that high-anxiety smokers might be affected more by the condition. This is also the case for cravings for smoking. Cravings increased after stress conditions in both groups, but the reactions to control conditions were different. Cravings of low-anxiety smokers slightly increased, but those of high-anxiety smokers decreased even more after control conditions, creating a bigger difference in cravings according to the types of conditions.

**Table 3:** Self-reported measurements before and after conditions.

	**High-anxiety**		**Low-anxiety**		**Test statistics (*F*)**
	**Stress (*****n** =* **30)**	**Control (*****n** =* **30)**	**Stress (*****n** =* **30)**	**Control (*****n** =* **30)**	
Change in STAI-X-1	3.00 (5.78)	−6.23 (8.21)	2.30 (7.89)	−2.33 (5.07)	
Anxiety					1.63
Stress					30.55^***^
Anxiety × Stress					3.36
Change in Craving-VAS	11.97 (17.66)	−9.90 (15.55)	17.03 (15.16)	2.57 (18.70)	
Anxiety					8.14^**^
Stress					34.96^***^
Anxiety × Stress				1.45

Mean (standard deviation); STAI-X-1, State-Trait Anxiety Inventory; Craving-VAS, Craving-Visual Analog Scale; Test Statistics (*F*), results of the two-way ANOVA. ^**^p < 0.01. ^***^p < 0.001.

#### 3.3.2 Gaze patterns

In order to examine the differences in gaze patterns between the two groups in terms of types of conditions, a 2 (time: before, after) × 2 (anxiety: high, low) × 2 (condition: stress, control) three-way mixed ANOVA was conducted with one within-subjects factor and two between-subjects factors.

**Figure 4:**
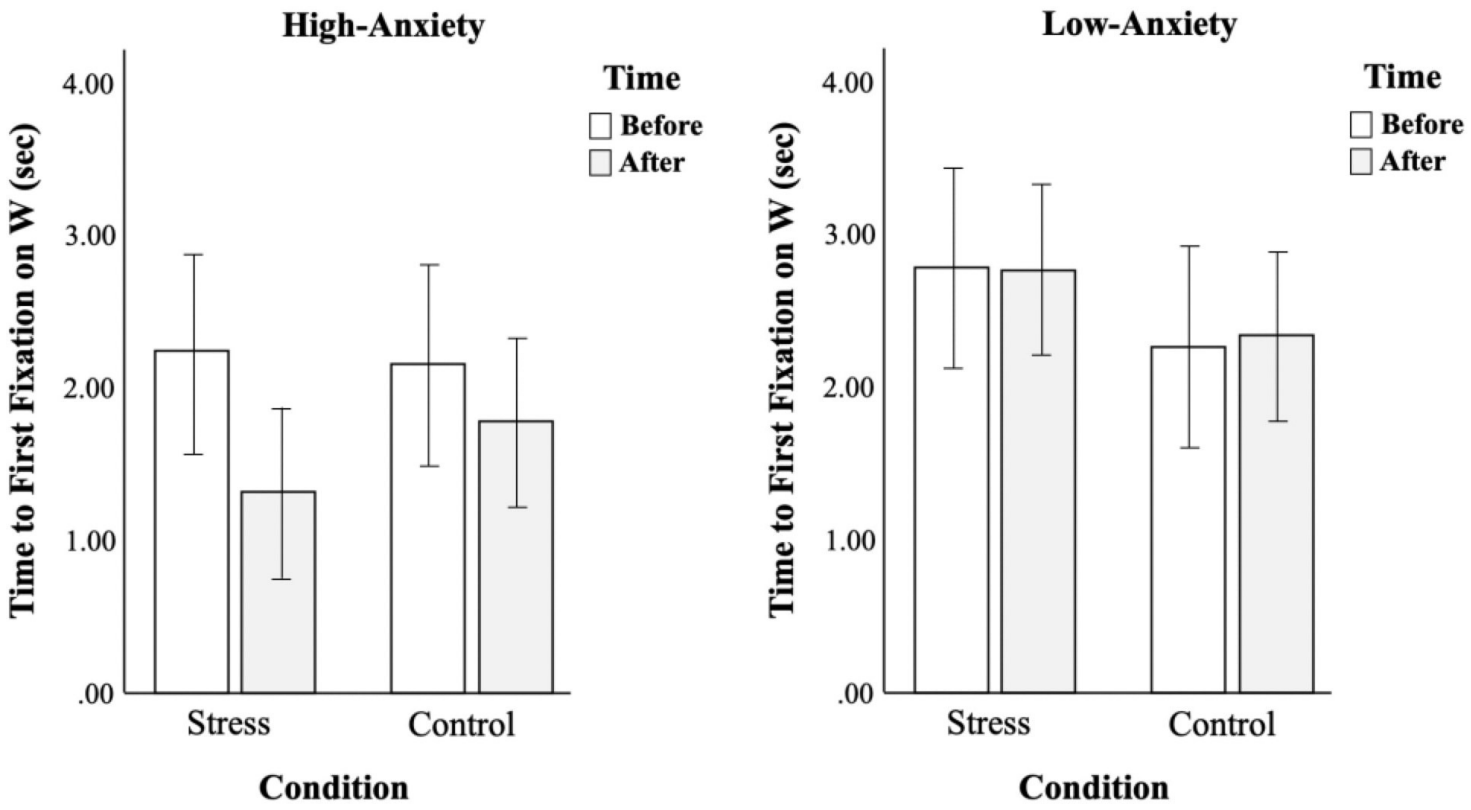
Comparison of changes in time to first fixation on warnings before and after conditions between two groups.

For the time to first fixation, there was a significant interaction between time and anxiety [*F*_(3,116)_ = 4.04, *p* < 0.05, $\eta_{p}^{2}$ = 0.03], but no other significant effects (see Table 4). As shown in Figure 4, time to first fixation of low-anxiety smokers on warnings stays almost the same whereas that of high-anxiety smokers decreases, more drastically under stress conditions. In other words, gaze patterns of low-anxiety smokers in terms of time to first fixation on warnings were not affected by types of conditions, but those of high-anxiety smokers were affected and high-anxiety smokers showed the tendency to fixate on warnings faster in the second trial.

**Table 4:** Gaze patterns before and after conditions.

	**High-anxiety**		**Low-anxiety**		**Test statistics (*F*)**
	**Stress (*****n** =* **30)**	**Control (*****n** =* **30)**	**Stress (*****n** =* **30)**	**Control (*****n** =* **30)**	
**Time to first fixation on W (sec)**					
Before	2.23 (2.02)	2.15 (1.57)	2.77 (1.79)	2.25 (1.89)	
After	1.32 (1.25)	1.78 (1.17)	2.76 (1.78)	2.32 (1.82)	
Time					3.34
Time × Anxiety					4.04^*^
Time × Stress					0.86
Time × Anxiety × Stress					0.47
**Fixation duration on W (%)**					
Before	44.04 (27.13)	47.47 (16.68)	37.60 (19.95)	41.45 (23.13)	
After	57.50 (19.44)	46.69 (14.36)	39.03 (16.94)	47.86 (22.80)	
Time					8.84^**^
Time × Anxiety					0.49
Time × Stress					1.80
Time × Anxiety × Stress					7.75^**^

Mean (standard deviation); Fixation Duration on W (%), fixation duration on warnings/(fixation duration on warnings + fixation duration on branding); Test Statistics (*F*), results of the three-way mixed ANOVA. ^*^p < 0.05. ^**^p < 0.01.

**Figure 5:**
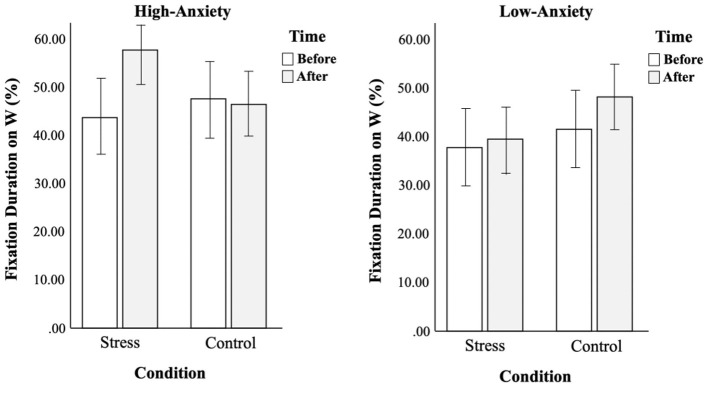
Comparison of changes in fixation duration on warnings before and after conditions between two groups.

For the fixation duration, there was a significant three-way interaction between factors [*F*_(3,116)_ = 7.75, *p* < 0.01, $\eta_{p}^{2}$ = 0.06] (see Table 4). As shown in Figure 5, gaze patterns were opposite between the two groups. High-anxiety smokers did not show much difference in fixation duration on warnings under control conditions, but they spent more time looking at warnings after stress conditions. On the other hand, low-anxiety smokers spent more time looking at warnings under control conditions, but not under stress conditions.

### 3.4 Exploratory analysis

As the results of this study differed from expected, additional analysis was conducted to have a better insight into these results.

#### 3.4.1 Gaze patterns

**Figure 6:**
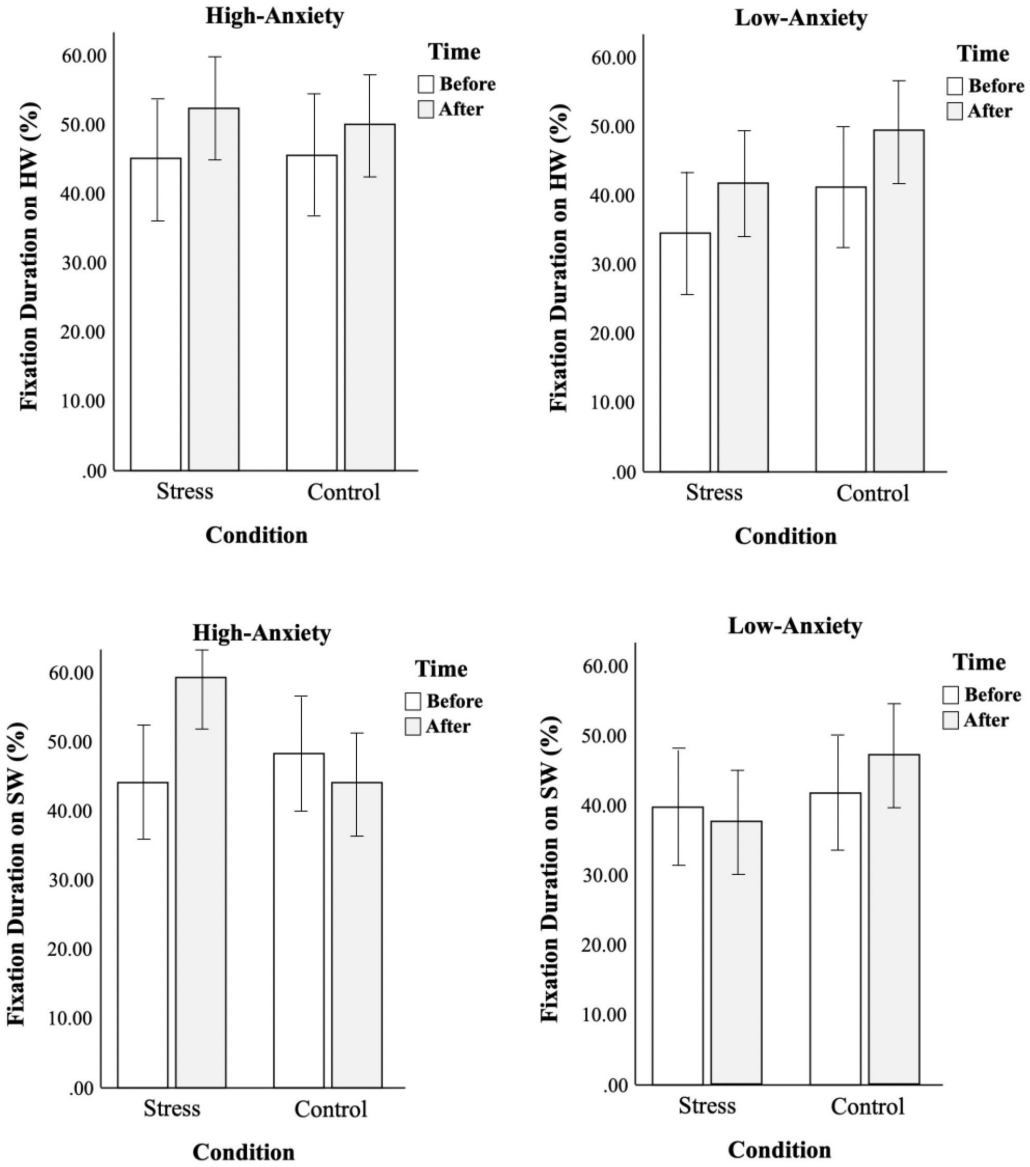
Comparison of fixation durations on health- vs. social-focused warnings before and after conditions between two groups.

In order to understand the contributing factor of the opposite gaze patterns that were seen between high-anxiety and low-anxiety smokers, the fixation durations on different types of warnings were analyzed separately. The same analysis of three-way mixed ANOVA as the main analysis was conducted. The analysis of Korean warnings vs. foreign warnings did not disclose anything discriminatory, but the analysis of health-focused warnings vs. social-focused warnings revealed where the main results came from. As shown in Table 5 and Figure 6, all smokers allocated more visual attention to health-focused warnings for the second trial while the same gaze patterns as the main analysis were seen in the visual attention to social-focused warnings. This tells us that social-focused warnings were the main source of the opposite gaze patterns between high-anxiety and low-anxiety smokers.

**Table 5:** Gaze patterns on different types of warnings.

	**High-anxiety**		**Low-anxiety**		**Test statistics (*F*)**
	**Stress (*****n** =* **30)**	**Control (*****n** =* **30)**	**Stress (*****n** =* **30)**	**Control (*****n** =* **30)**	
**Fixation duration on HW (%)**					
Before	44.90 (29.16)	45.64 (16.95)	34.23 (24.32)	40.93 (25.18)	
After	52.39 (21.43)	49.66 (15.43)	41.39 (17.78)	48.75 (26.39)	
Time					9.68^**^
Time × Anxiety					0.17
Time × Stress					0.11
Time × Anxiety × Stress					0.24
**Fixation duration on SW (%)**					
Before	44.09 (27.56)	48.18 (20.86)	39.75 (19.97)	41.68 (22.34)	
After	59.21 (20.66)	43.74 (17.63)	37.61 (18.79)	47.26 (24.36)	
Time					3.13
Time × Anxiety					0.82
Time × Stress					2.19
Time × Anxiety × Stress					11.68^**^

Mean (standard deviation); HW, Health-focused Warnings; SW, Social-focused Warnings; Test Statistics (*F*), results of the three-way mixed ANOVA. ^**^p < 0.01.

As the fixation duration on warnings was not significantly different in terms of the level of anxiety, fixation durations were divided into groups in ascending order so that one-way ANOVA could be conducted on other variables from self-reported measurements except BAI.

The fixation durations of the first trial were divided into three groups: the least-fixation group that spent 0 percent to 36 percent of the time on warnings (*n* = 39), the mid-fixation group that spent 36 percent to 51 percent of the time (*n* = 40), and the most-fixation group that spent 51 percent to 100 percents of the time (*n* = 41). This analysis discovered one significant result. The scores of PPFI were the highest in the least-fixation group [M(SD) = 47.00(7.82)], the middle in the mid-fixation group [M(SD) = 45.70(8.08)], and lowest in the most-fixation group [M(SD) = 41.10(10.66)] with its significant effect [*F*_(2,117)_ = 4.82, *p* < 0.05, $\eta_{p}^{2}$ = 0.08]. The more psychological flexibility resulted in less time on warnings.

In order to see the effect of PPFI in terms of the presence of emotional distress, the changes in fixation durations before and after conditions were also divided into groups. Unlike the main analysis that used repeated measure analysis, the changes in fixation durations were calculated in the same way that changes in STAI-X-1 and Craving-VAS were calculated. Groups were divided based on the tendency to fixate more or less on warnings after conditions. There were 68 smokers who fixated on warnings more after conditions [*n*(stress) = 38, *n*(control) = 30] and 52 smokers who fixated on warnings less after conditions [*n*(stress) = 22, *n*(control) = 30]. A 2 (condition: stress, control) × 2 (attention allocation: warning, branding) two-way ANOVA was conducted with the dependent variable of PPFI. The result showed that there was a significant interaction [*F*_(3,116)_ = 8.46, *p* < 0.01, $\eta_{p}^{2}$ = 0.07]. Smokers under stress conditions had similar scores of psychological flexibility whether they have attentional allocations to warning or branding, but under control conditions, smokers who had attentional allocations to warnings had much higher psychological flexibility. Although more psychological flexibility resulted in less time on warnings for the first encounter with warnings, more psychological flexibility resulted in more time on warnings for the repeated encounter with warnings when not under stress conditions.

### 3.5 Summary

The study concludes that the level of anxiety did not affect visual attention in the way that was expected. High-anxiety smokers showed a stronger motive of managing negative affect with smoking, less confidence in controlling their smoking cravings, greater avoidance of pain or discomfort when pursuing life goals, and greater reactance toward GHWs, but these characteristics of high-anxiety smokers did not reflect the attentional behaviors toward GHWs. There was no difference in the time to the first fixation on warning or branding, and the fixation duration on warnings between high-anxiety and low-anxiety smokers. Stress-inducing conditions did work as they could increase state anxiety and smoking cravings, but only cravings increased significantly more in high-anxiety smokers. After stress conditions, high-anxiety smokers showed less time to first fixate on warnings, unlike low-anxiety smokers. Fixation durations of high-anxiety smokers increased after stress conditions while those of low-anxiety smokers increased after control conditions. Further analysis discovered that the same gaze patterns were seen only on social-focused warnings. Moreover, instead of anxiety level, psychological flexibility seemed to affect the visual attention to warnings, for the higher flexibility the greater visual attention to warnings. There was also no evidence of significantly higher distress intolerance in high-anxiety smokers. Therefore, high-anxiety smokers showed avoidance behaviors in life, but when treating cigarette packs they did not show avoidance toward health warnings.

## 4 Discussion

The current study examined how smokers with higher levels of anxiety are different from those with lower levels of anxiety in characteristics and attentional bias toward cigarette warnings, especially in response to emotional distress.

The results of the first measurement of the eye-tracking task without the effect of emotional distress demonstrated that there was no difference between high-anxiety smokers and low-anxiety smokers in attentional bias toward cigarette warnings. No significant results could be attributed to the fact that they are repeatedly and regularly exposed to the stimuli used in this experiment. Even though stimuli included foreign warnings, the messages were repetitive enough to have no discriminating effect. This result contradicts the hypervigilance-avoidance hypothesis that individuals with higher levels of anxiety show faster detection of threats and then later avoidance (Mogg et al., 2004). Later attentional avoidance is considered to be a strategy that individuals with anxiety use to manage emotions, which is a part of cognitive control (Cisler and Koster, 2010). Nevertheless, not only significantly faster detection of warnings but also later avoidance was not seen in high-anxiety smokers.

The effect of emotional distress had a different influence on smokers. In terms of time to first fixation, low-anxiety smokers did not show much difference before and after both conditions, but high-anxiety smokers fixated on warnings faster after both conditions. Although high- and low-anxiety smokers did not show a significant difference in how fast they detected the threatening stimuli for the first viewing, their difference got greater after condition-forming stages and got greatest under stress conditions. This kind of gaze pattern is known as negativity bias or selective attention to the threat that people with anxiety have the increased attention on negative or threatening stimuli (Mogg and Bradley, 1998). The familiarity with the threatening stimuli of cigarette warnings could have suppressed negativity bias, or even enacted the avoidance in hypervigilance-avoidance hypothesis, but stress conditions might have triggered them to be in the hypervigilant stage again.

For fixation duration on warnings, high-anxiety and low-anxiety smokers demonstrated the opposite gaze pattern. Fixation durations of high-anxiety smokers increased under stress conditions whereas those of low-anxiety smokers increased under control conditions. The exploratory analysis discovered that these gaze patterns came from the fixation durations on social-focused warnings. All smokers fixated longer on health-focused warnings regardless of the types of conditions. These gaze patterns can be explained by the effect of repeated displays of cigarette packs. Participants reported that at the first trial of the eye-tracking task they put their attention on brandings as they normally would to find out the information about the cigarettes, such as the amount of nicotine in mg and the name of the cigarettes. However, at the second trial, after gaining enough information on cigarettes, they distributed their attention to other parts, and in this case, the only option was warnings. Others reported that they were resistant to looking at the warnings at first, but as cigarettes were displayed continuously, they felt compelled to see the warnings.

As seen in the results, increased visual attention to health-focused warnings for the second trial was consistent with participants' reports, but social-focused warnings were treated differently by smokers. Low-anxiety smokers distributed their attention to social-focused warnings like they did with health-focused warnings under control conditions, but they were more resistant to look at social-focused warnings under stress conditions. This result underlines that low-anxiety smokers become more avoidant toward social-focused warnings when they are under stress because in the experiment stress came from the social situation and not from a health issue. This is in line with a previous study that when people are not in control of the threat their attention decreases whereas it increases when people are in control of the threat (Notebaert et al., 2020) as was observed among low-anxiety smokers under control conditions. This is a protective response of low-trait anxious people that allocates attentional resources away from the threatening stimuli (Williams et al., 1988).

On the other hand, anxious smokers fixated on social-focused warnings much longer under stress conditions while they fixated less under control conditions. This can be again explained by hypervigilance-avoidance or delayed disengagement from threat. After the activation of faster detection on threats by hypervigilance, they might have stayed in that stage under stress conditions, as anxiety is associated with slower attentional disengagement from threats (Fox et al., 2001), which increased overall fixation durations. However, under control conditions, they could have moved to the avoidance stage where they avoid the type of threat that gives the most discomfort or negative affect. Overall, the results show that smokers were more affected by social-focused warnings than health-focused warnings, thus behave differently according to the circumstances.

According to the self-reported measurements, high-anxiety smokers had a stronger motive to manage negative affect with smoking, less confidence in controlling smoking urges, greater avoidance toward pain or discomfort in life, and more reactance toward graphic health warnings compared to low-anxiety smokers as expected. However, they did not differ in nicotine dependence, intention to quit, or awareness of the consequences of smoking. They are well aware of how smoking will affect their health but have reactance toward health warnings because they are less confident in controlling smoking urges especially in the presence of negative affect. The measurements on awareness of the consequences of smoking and psychological reactance proceeded lastly after the second trial of the eye-tracking task. Regardless of the hypervigilance toward warnings, meaning more fixation duration, high-anxiety smokers had a greater reactance. This tells us that the conflict between their avoidant behavior and hypervigilance might have resulted in more reactance and the increased fixation duration does not necessarily mean the efficacy of cigarette warnings.

This study anticipated that the increase in state anxiety and cravings under stress conditions would lead to smokers' greater avoidance of GHWs, but the results of visual attention toward cigarette warnings showed that the attention was not directly affected by state anxiety or cravings. However, the relationship between state anxiety and cravings emerged when they were put together. When the state anxiety of high-anxiety smokers increased, their cravings also increased, but when their anxiety decreased, their cravings also decreased, suggesting that the cravings of high-anxiety smokers are connected with state anxiety. On the contrary, when the anxiety of low-anxiety smokers dropped under control conditions, their cravings increased instead, suggesting that the energy consumption by the use of cognition in the task is more connected with cravings for low-anxiety smokers.

Although there was no significant interaction effect for state anxiety, the breadth of change in state anxiety according to the types of conditions within the high-anxiety group was bigger than that within the low-anxiety group. In the control condition, the state anxiety of high-anxiety smokers decreased much more than those of low-anxiety smokers. This implies that when uncertainty about the experimental tasks that were not anxiety-provoking was resolved, the subjective feeling of having anxiety might have dropped more drastically in high-anxiety people compared to low-anxiety people for anxiety being defined as an anticipatory response to uncertainty (Grupe and Nitschke, 2013). This result indicates that high-anxiety smokers may be more sensitive to the circumstance and influenced by it.

Distress intolerance was not seen in high-anxiety smokers, but the performance was significantly lower. This study was not designed to guarantee a certain period of time for the experiment. The faster participants finish the experiment, the earlier they could leave. Although they were informed that the experiment would take about 40 min beforehand, they were not instructed to stay for 40 min however fast they finished the experiment. Therefore, those who want to get out fast would choose to quit faster, not just because they could not stand the distress involved with the task. Performance on the stress-inducing task, however, was affected by the level of anxiety, which means that high-anxiety people perform worse under stress or with the stress-inducing task.

Further analysis implied that psychological flexibility to endure discomfort rather than the level of anxiety might have more impact on gaze patterns. The results on psychological flexibility are noteworthy since they appear to be conflicting. For the fixation duration of the first trial, the result showed that the more psychological flexibility, the lesser time on warnings. However, more psychological flexibility was associated with the increased fixation duration on warnings under control conditions after the repeated trial. These discoveries demonstrate that smokers with more psychological flexibility do not show hypervigilance toward the threatening stimuli for the first encounter and satisfy their goal of searching for the necessary information on cigarettes, but when the threatening stimuli are repeatedly displayed and their goal was fulfilled, they show acceptability toward disturbing stimuli and are less avoidant toward them. This result is more consistent with the results of fixation durations of low-anxiety smokers on social-focused warnings where they show more attention to warnings when they feel in control. These findings reflect more clearly the definition of psychological flexibility as the ability to respond flexibly to situations in a way that facilitates the pursuit of goals despite pain or discomfort (Hayes et al., 2004; Kashdan et al., 2020). In this study, psychological flexibility is not what encourages to relinquish avoidant behavior that helps to maintain substance use, but what enables smokers less affected by warnings.

The limitation of this study is the unusualness of cigarette-viewing circumstances. Smokers do not normally experience viewing 8 cigarette packs in a row twice that are displayed in front of their eyes with the instruction of not moving their head for accurate recordings by the eye-tracking device and without any other options to see. Moreover, only the fronts of cigarette packs were displayed, but participants reported that the sides of cigarette packs are the areas they mostly look at because they are where all the information is. Future studies would benefit if all sides of cigarettes were available for participants to see. Another limitation would be the age of the participants. Most of the participants were college students. They would be less affected by health-focused warnings because they are less concerned with health. Instead, young smokers might be more affected by social-related issues. The results of this study might have been different if this study included much older participants who may have more concerns about being sick from smoking. Furthermore, the impact of more attention to warnings due to stress conditions on the smoking behaviors of high-anxiety smokers has yet to be investigated.

This study explored how the characteristics of high-anxiety smokers are associated with visual attention to health warnings, especially avoidance patterns that high-anxiety smokers have. These results demonstrated that different characteristics of smokers could affect how they viewed cigarette warnings. High-anxiety smokers may have been in a state of avoidance regarding cigarette warnings that they were repeatedly exposed to, but emotional distress triggers them to activate a state of hypervigilance and fixate more on warnings they consider as threats. Low-anxiety smokers with more psychological flexibility can allocate attention to warnings as acceptability of discomfort is intact, but under stress, they show avoidance toward them to protect themselves from disturbing stimuli. The results implied that high-anxiety smokers are more vulnerable to emotional distress and the effectiveness of cigarette warnings may differ depending on not only smokers' characteristics but also the circumstances. In spite of the increased fixation duration due to hypervigilance, the effectiveness of cigarette warnings may be limited as they had more psychological reactance toward them. Furthermore, the result that showed no significant difference in time to first fixation and fixation duration on between Korean and foreign warnings tells us that change of warning pictures every 24 months by the regulation of Ministry of Health and Welfare (2016) with the same types of messages may not be as effective as desired. Therefore, in order to increase the effectiveness, more diverse types of cigarette warnings could be applied to target more wide range of smokers, such as warnings that are gain-framed (Kim and Moon, 2017; Sillero-Rejon et al., 2021). This is consistent with a traditional norm of encoding variability theory (Hintzman, 1974) supporting the effectiveness of variation in contents and intervals. High-anxiety smokers could benefit from these findings by understanding that psychological reactance toward the idea of quitting could be strengthened by hypervigilance under stress but this bodily reaction is not to be confused with one's real input on quitting, while healthcare providers could also benefit by explaining this mechanism to reduce psychological reactance toward health warnings. Furthermore, high-anxiety smokers would benefit from intervention programs such as Anxiety Sensitivity Reduction Program for Smoking (ASRP-S) that provides the psychoeducational rationale of the negative bi-directional relationship between anxiety and smoking behaviors and helps to better manage emotional distress and smoking behaviors (Redmond et al., 2024).

## Data Availability

The raw data supporting the conclusions of this article will be made available by the authors, without undue reservation.
